# Differential Requirement for Utrophin in the Induced Pluripotent Stem Cell Correction of Muscle versus Fat in Muscular Dystrophy Mice

**DOI:** 10.1371/journal.pone.0020065

**Published:** 2011-05-16

**Authors:** Amanda J. Beck, Joseph M. Vitale, Qingshi Zhao, Joel S. Schneider, Corey Chang, Aneela Altaf, Jennifer Michaels, Mantu Bhaumik, Robert Grange, Diego Fraidenraich

**Affiliations:** 1 Department of Cell Biology and Molecular Medicine, University of Medicine and Dentistry of New Jersey, New Jersey Medical School, Newark, New Jersey, United States of America; 2 Department of Neurology, University of Medicine and Dentistry of New Jersey, New Jersey Medical School, Newark, New Jersey, United States of America; 3 Department of Pediatrics, University of Medicine and Dentistry of New Jersey, Robert Wood Johnson Medical School, Piscataway, New Jersey, United States of America; 4 Department of Human Nutrition, Foods and Exercise, Virginia Polytechnic Institute and State University, Blacksburg, Virginia, United States of America; Johns Hopkins University School of Medicine, United States of America

## Abstract

Duchenne muscular dystrophy (DMD) is an incurable degenerative muscle disorder. We injected WT mouse induced pluripotent stem cells (iPSCs) into mdx and mdx∶utrophin mutant blastocysts, which are predisposed to develop DMD with an increasing degree of severity (mdx <<< mdx∶utrophin). In mdx chimeras, iPSC-dystrophin was supplied to the muscle sarcolemma to effect corrections at morphological and functional levels. Dystrobrevin was observed in dystrophin-positive and, at a lesser extent, utrophin-positive areas. In the mdx∶utrophin mutant chimeras, although iPSC-dystrophin was also supplied to the muscle sarcolemma, mice still displayed poor skeletal muscle histopathology, and negligible levels of dystrobrevin in dystrophin- and utrophin-negative areas. Not only dystrophin-expressing tissues are affected by iPSCs. Mdx and mdx∶utrophin mice have reduced fat/body weight ratio, but iPSC injection normalized this parameter in both mdx and mdx∶utrophin chimeras, despite the fact that utrophin was compromised in the mdx∶utrophin chimeric fat. The results suggest that the presence of utrophin is required for the iPSC-corrections in skeletal muscle. Furthermore, the results highlight a potential (utrophin-independent) non-cell autonomous role for iPSC-dystrophin in the corrections of non-muscle tissue like fat, which is intimately related to the muscle.

## Introduction

Duchenne muscular dystrophy (DMD) is an X-linked degenerative disorder of muscle that affects 1 in 3500 newborn males. Progressive muscle weakness generally begins in preschool years. Affected boys are confined to wheelchairs in adolescence, and mortality in young adulthood results from cardiomyopathy or respiratory failure [1], [2].

Mutations are found in the gene for a 427 Kd dystrophin protein, which localizes to the sarcolemma of the skeletal muscle. Dystrophin is the key protein in the dystrophin-glycoprotein complex (DGC), which includes dystroglycans, sarcoglycans, dystrobrevin and syntrophin [3]. In the absence of dystrophin, the DGC is unstable and destroyed. Absence of the DGC results in mechanically induced damage of the skeletal muscle fibers [4], [5], [6].

The mdx mice bear a naturally occurring mutation in exon 23 of the dystrophin gene that abrogates expression of the full-length dystrophin protein, causing symptoms of muscular dystrophy [7], [8]. These mice exhibit moderate signs of skeletal muscle dystrophy but have nearly normal lifespan. The dystrophin-related protein utrophin is up-regulated in the sarcolemma of mdx muscle [9], [10], [11]. Mutations in both the dystrophin and the utrophin genes result in a more severe phenotype and premature death [10], [11]. Conversely, activation of utrophin or introduction of utrophin in mdx by transgenesis or viral delivery produces significant recoveries [11], [12], [13], [14], [15].

Several therapeutic strategies for treatment of DMD have been investigated [16], [17], [18], [19], [20], [21], [22], [23], [24], [25], [26], [27]. Induced pluripotent stem cells (iPSCs) are an attractive source for cell-based therapy, in part because human iPSCs can be reprogrammed from adult fibroblasts of patients [28], [29], [30], [31], [32], [33]. Previous studies in mice show that only a fraction of the muscle must express full-length dystrophin to confer protection against the development of DMD, as mice with a mosaic pattern of expression in the skeletal muscle displayed a markedly milder phenotype than mdx mice [34]. To create a mosaic model of DMD, we injected WT embryonic stem cells (ESCs) into mdx blastocysts [35]. ESCs incorporated at a low percentage to supply sarcolemma-dystrophin and to effect corrections. We also injected mdx ESCs into blastocysts. ESCs incorporated but failed to supply dystrophin and did not effect corrections [35]. Recently, mdx iPSCs and ESCs containing an artificial chromosome with a full-length human dystrophin gene were injected into mdx blastocysts to supply human dystrophin to muscle and heart, but corrective analyses of the muscle were not performed [36], [37]. Here we injected iPSCs into mdx single mutant and mdx∶utrophin double mutant blastocysts, to generate a mosaic rescue paradigm and to study the corrective potential of the iPSCs in mild and severe mouse models of DMD. We show that the iPSCs supply dystrophin to the sarcolemma to effect corrections in the skeletal muscle of mdx but not mdx∶utrophin. However, the iPSCs produce corrections of fat mass in both.

## Results

### iPSCs incorporate globally

**Figure 1:**
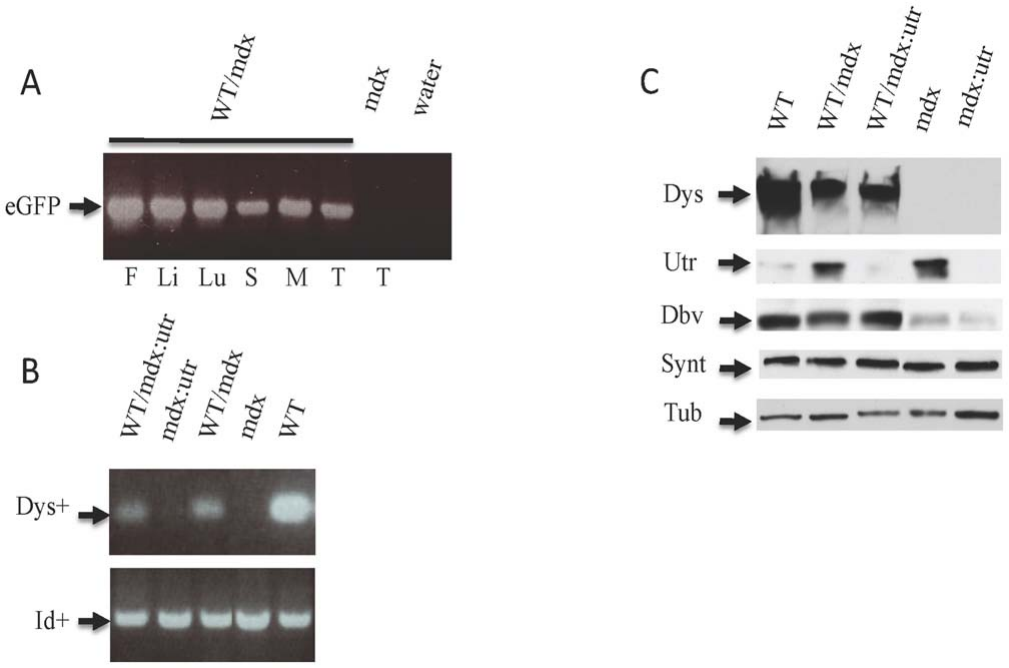
iPSCs incorporate globally. a) PCR analysis showing that eGFP marker (indicative of iPSCs) is present globally in the WT/mdx chimera; b) Semi Q genomic PCR using DNA from quadriceps muscle. Note that the intensity of the Dys+ band (indicative of percentage of iPSC chimerism) in WT/mdx and WT/mdx∶utrophin is approximately 1/5 of that observed in WT; c) WB analysis showing dystrophin, utrophin, dystrobrevin and syntrophin production in the quadriceps. Abbreviations: eGFP: enhanced green fluorescent protein, F: fat, Li: liver, Lu: lung, S: spleen, M: quadriceps muscle, T: tail, dys+: WT allele of dystrophin, Id+: WT allele of Id1 control (Id1 is present in both iPSC-derived and blastocyst-derived cells, and thus, is invariant among WT, KO and chimeric groups), Dys: 420 Kd dystrophin, Utr: 420 Kd utrophin, Dbv: 40 Kd (isoform of the dystrophin-dependent) dystrobrevin-alpha, Synt: 55 Kd syntrophin; Tub: 55 Kd tubulin.

**Figure 2:**
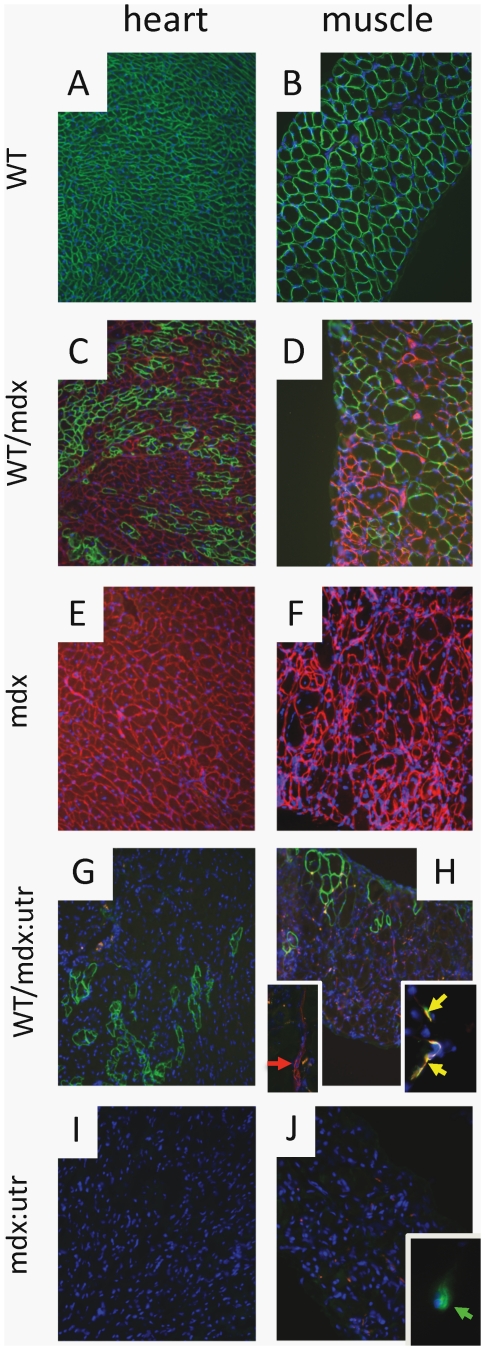
Dystrophin is supplied to chimeric muscle. WT (A, B), WT/mdx (C, D) and WT/mdx∶utrophin (G, H) heart and diaphragm muscle display sarcolemma-dystrophin (green staining); WT/mdx (C, D) and mdx (E, F) heart and diaphragm muscle display sarcolemma-utrophin (red staining); red arrow: utrophin-positive axon (H inset left); yellow arrows: utrophin-positive (red)/alpha-BT-positive (green) NMJ (H inset right); green arrow: utrophin-negative/alpha-BT-positive (green) NMJ (J inset); blue: nuclear staining (DAPI). Magnification: 100×; H inset left and right: 200× and 400× respectively; J inset: 400×.

We injected mouse WT eGFP-marked iPSCs into mdx, mdx∶utrophin+/− and mdx∶utrophin−/− blastocysts. We produced WT/mdx, WT/mdx∶utrophin+/− and WT/mdx∶utrophin−/− chimeric mice. WT/mdx and WT/mdx∶utrophin+/− chimeras displayed an identical phenotype, and thus, both genotypes were grouped under the name of WT/mdx. WT/mdx∶utrophin−/− chimeras displayed a distinct phenotype from that of WT/mdx and thus, were named WT/mdx∶utrophin. A total of 10 WT/mdx and 4 WT/mdx∶utrophin mice in the range of 10–30% mosaicism were used in the study. At 1 week of age, chimeric pups were identified by performing genomic PCR of the eGFP transgene from DNA of tail biopsies (data not shown). After sacrifice (10–12 weeks for WT/mdx∶utrophin and 12–15 weeks for WT/mdx), global incorporation was assessed by performing PCR of the eGFP transgene in fat, liver, lung, spleen, muscle (quadriceps, pectoralis, diaphragm), tail and heart (Fig. 1A and data not shown). The degree of chimerism was determined by performing semi-quantitative genomic PCR of the WT allele of dystrophin using DNA samples from skeletal muscle, heart and tail (Fig. 1B for skeletal muscle and data not shown). Unlike the skeletal muscle, the cardiac muscle does not syncytialize, and therefore, the percentage of dystrophin-positive cardiac myocytes represents an accurate measure of the degree of chimerism [35]. Thus, the degree of chimerism was further confirmed by immunofluorescence (IMF) for dystrophin in heart sections (Fig. 2) [35]. Dystrophin detection in skeletal muscle was also verified by dystrophin IMF (Fig. 2 for diaphragm and data not shown for quadriceps and pectoralis).

### Dystrophin is supplied to the chimeric muscle

The iPSCs incorporated into the heart and skeletal muscle of WT/mdx and WT/mdx∶utrophin chimeras and supplied dystrophin (Fig. 1c and Fig. 2). As expected, the presence of sarcolemma-utrophin was observed in the dystrophin-negative fibers of WT/mdx but not in WT/mdx∶utrophin muscle (Fig. 2). No utrophin was detected in dystrophin-positive (iPSC-derived) fibers in WT/mdx (Fig. 2). Consistent with lack of sarcolemma-utrophin upregulation in WT/mdx∶utrophin muscle (Fig. 2), negligible utrophin levels were detected by WB (Fig. 1c). As expected, utrophin was detected in a portion of the neuromuscular junction (NMJ) and the axons of WT/mdx∶utrophin muscle (Fig. 2H, insets).

**Figure 3:**
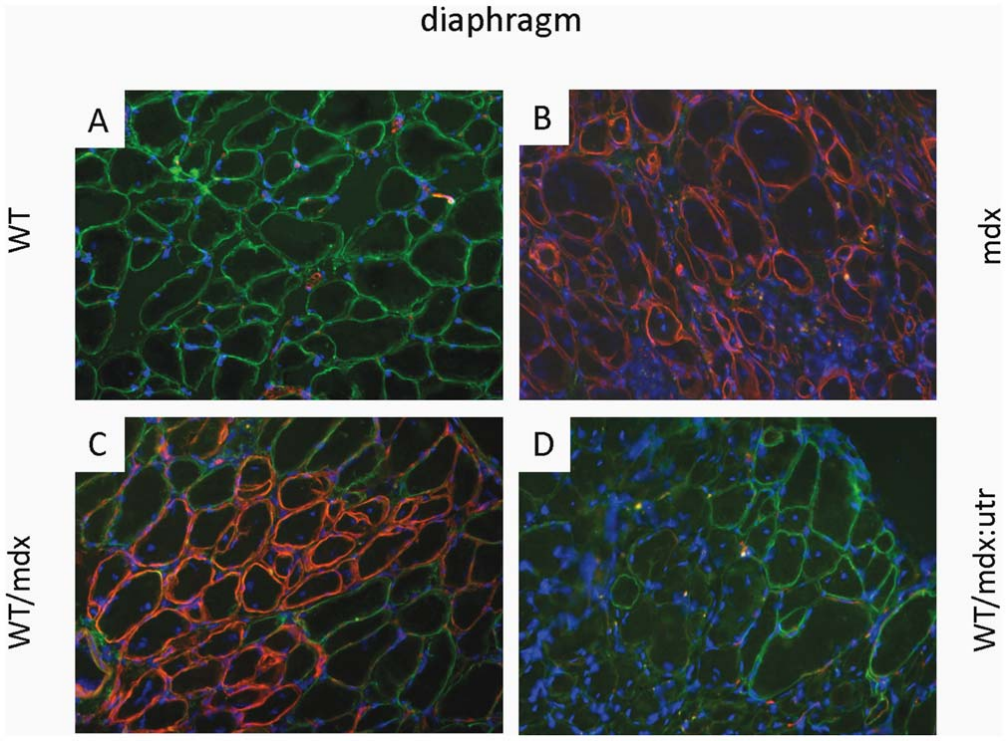
Dystrobrevin is supplied to chimeric muscle. WT (A), WT/mdx (C) and WT/mdx∶utr (D) diaphragm muscle display sarcolemma-dystrobrevin (green staining); mdx (B) and WT/mdx (C) diaphragm muscle display sarcolemma-utrophin (red staining); blue: nuclear staining (DAPI); magnification: 200×.

**Figure 4:**
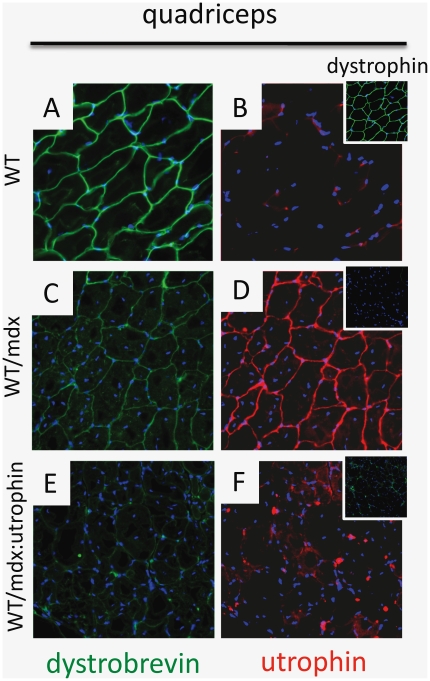
Utrophin sustains weak dystrobrevin staining. Dystrobrevin (A) is detected in WT quadriceps muscle; weak dystrobrevin (C) and utrophin (D) are detected in WT/mdx quadriceps muscle areas with undetectable dystrophin (D inset); no dystrobrevin (E) and no utrophin (F) are detected in WT/mdx∶utrophin quadriceps muscle areas with undetectable dystrophin (F inset); Abbreviation: utr: utrophin; blue: nuclear staining (DAPI); magnification: 200×.

iPSC incorporation increased staining of dystrobrevin (Dbv) (Fig. 1c), which was found at the sarcolemma of the skeletal muscle (Fig. 3C and D). Weak sarcolemma-dystrobrevin staining was observed in areas of undetectable dystrophin (utrophin-positive) in mdx but not in mdx∶utrophin chimeric muscle (Fig. 4C, compare with 4E). Consistent with co-localization of dystrobrevin and utrophin, a residual dystrobrevin band was more intense in mdx than mdx∶utrophin muscle (Fig. 1C). Syntrophin was not affected by absence of dystrophin or utrophin (Fig. 1C). Thus, the iPSCs incorporate in the mdx and mdx∶utrophin muscle and supply dystrophin to the sarcolemma.

### Functional corrections

We determined the functional consequences of WT iPSC incorporation in mdx muscle. To this end, extensor digitorum longus (EDL) muscle was isolated and subjected to stress tests (Table 1). Stress output (force/cross sectional area) for tetanic and twitch contractions from the EDL of WT/mdx were comparable to that of WT and (ESC-derived) WT/mdx mice [35]. Thus, both ESCs and iPSCs effect functional corrections in mdx muscle.

**Table 1:** Contractile stress recovery in WT/mdx EDL muscle.

Genotype	tetanus (g/mm^2^)	twitch (g/mm^2^)
WT	48.4±2.0	7.2±0.3
WT/mdx*	43.9±1.0	6.5±0.1
Mdx	24.4±7.3	3.2±0.3

*P<0.05 relative to mdx.

### Histological corrections

**Figure 5:**
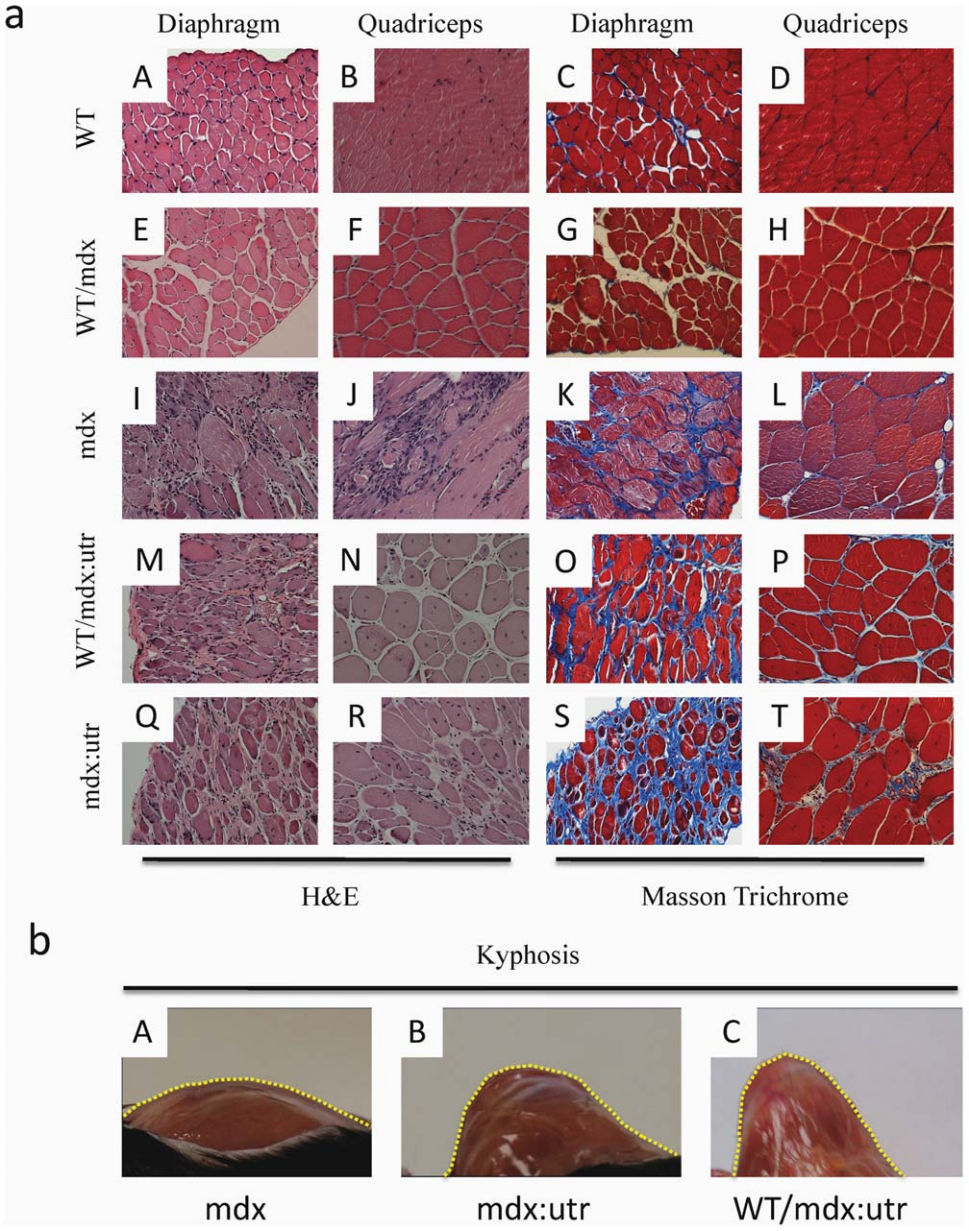
WT/mdx but not WT/mdx∶utrophin mice show improved muscle pathology. a) H&E staining of diaphragm and quadriceps muscles of WT (A, B), WT/mdx (E, F), mdx (I, J), WT/mdx∶utrophin (M, N) and mdx∶utrophin (Q, R) mice; Masson trichrome staining of diaphragm and quadriceps muscles of WT (C, D), WT/mdx (G, H), mdx (K, L), WT/mdx∶utrophin (O, P) and mdx∶utrophin (S, T) mice; b) kyphosis in mdx∶utrophin (B) and WT/mdx∶utrophin (C) but not in mdx (A) mice; magnification: 200× (a) and 4× (b).

To study the effect of iPSC on histopathology, we analyzed the diaphragm, which develops fibrosis and mononuclear invasion (Fig. 5a) [8]. We also analyzed limb (quadriceps, Fig. 5a) muscles, which show central nucleation. WT and WT/mdx but not mdx muscle (diaphragm, quadriceps) show an organized architecture (fibers of similar size, Fig. 5aE), no central nucleation (<10%, Fig. 5aF), no mononuclear invasion (Fig. 5aE) and little or no fibrosis (Fig. 5aG). However, WT/mdx∶utrophin muscle (diaphragm, quadriceps) show a disorganized architecture (Fig. 5aM), increased central nucleation (>80%, Fig. 5aN), mononuclear invasion (Fig. 5aM) and extensive fibrosis though slightly diminished in comparison to mdx∶utrophin (Fig. 5aO). In support of a lack of correction observed in the absence of utrophin, the pathological curvature of the spine (kyphosis) observed in mdx∶utrophin mice [10] was still apparent in the WT/mdx∶utrophin chimeras (Fig. 5b). Thus, the iPSCs improve the pathology in the skeletal muscle of WT/mdx but not to the same extent in WT/mdx∶utrophin mice despite provision of dystrophin to both.

### Fat mass gain

**Figure 6:**
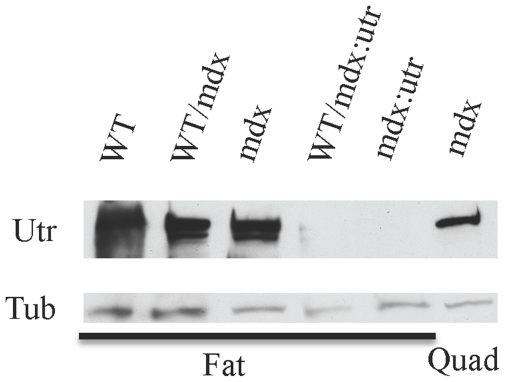
Utrophin is produced in the fat. WB analysis showing utrophin production in the fat of WT, WT/mdx and mdx mice. Utrophin production in the skeletal muscle of mdx is shown as control. Abbreviations: Utr: utrophin; Tub: tubulin; Quad: quadriceps muscle.

Other tissues were affected by the iPSCs. Mdx and mdx∶utrophin mice have reduced fat, but their fat was restored in the chimeras. Abdominal fat weight/body weight of WT/mdx mice (3.7±0.5%) or WT/mdx∶utrophin (4.1±0.6%) mice was comparable to that of WT mice (4.2±1.5%), and was improved relative to mdx (1.7±0.6%) or mdx∶utrophin mice (0.5±0.3%)(P<0.05). Utrophin was detected in the fat (Fig. 6). However, dystrophin was not (data not shown). In contrast to the ability of iPSCs to supply dystrophin to the skeletal muscle of mdx∶utrophin (Fig. 1C), the ability of the iPSCs to supply utrophin to the fat of mdx∶utrophin was compromised (Fig. 6). The results indicate that iPSCs effect corrections outside the muscle.

## Discussion

In this report we show that injection of mouse WT iPSCs at pre-implantation stage produces corrections in mouse models of muscular dystrophy. A rescue process in muscle and fat depends on the ability of the iPSCs to produce dystrophin. However, while the rescue in muscle appears to also depend on the presence of an intact utrophin gene, the rescue of fat does not.

### WT ESCs versus WT iPSCs: differentiation potential in a mutant environment

The iPSCs bear advantages over the ESCs, as human iPSCs can be reprogrammed from adult cells of human patients. We have previously shown that ESCs incorporate with widespread tissue distribution when injected into mdx blastocysts [35], [38], but the chimeric potential of the WT iPSCs when injected into a mutant blastocyst remained elusive. We show that a mutant environment does not compromise the capacity of the iPSCs to generate chimeras with widespread tissue distribution. Like the ESCs [35], the iPSCs bear a corrective potential.

### Corrections of skeletal muscle

Partial incorporation of dystrophin has been shown to correct the dystrophin-negative muscle, as mdx mice with a mosaic pattern of dystrophin expression manifest a milder phenotype [16], [18], [34], [39]. Furthermore, mosaic WT/mdx mice with a portion of ESC-derived muscle have improved pathology and function [35]. Here we tested iPSC injection, and report that iPSC-mosaic mice showed recovery in the skeletal muscle of WT/mdx but not of WT/mdx∶utr chimeras. In the mdx rescue, sarcolemma utrophin is detected in the iPSC-negative (dystrophin-negative) muscle fibers. As expected, dystrobrevin is present in iPSC-fibers, which contain high dystrophin. However, and consistent to previous results [35], dystrobrevin is also present in fibers containing low dystrophin (high utrophin) levels. Dystrobrevin may replace some of the functions of dystrophin to ultimately stabilize a portion of the DGC [6], [40]. It is possible that utrophin contributes to the stabilization of dystrobrevin in areas with low dystrophin in mdx chimeras. The notion of utrophin as a DGC stabilizer in chimeras is strengthened by our findings showing that a residual dystrobrevin band is more intense in mdx than in mdx∶utrophin muscle. This utrophin-dependent effect appears to be specific for some members of the DGC, as syntrophin levels are unaffected by loss of utrophin or dystrophin. The utrophin mutation in mdx∶utrophin chimeras appeared to affect the rescue process by the iPSCs, as the double mutant chimeras developed an abnormal fibrotic musculature.

Absence of sarcolemma-utrophin in the iPSC-fibers may reflect a process of inhibition of iPSC-utrophin by iPSC-dystrophin. On the other hand, the presence of iPSC-utrophin in NMJ indicates that the iPSCs are capable of producing utrophin. Thus, inhibition of iPSC-utrophin by iPSC-dystrophin appears to be sarcolemma-specific. The development of a fibrotic muscle in mdx∶utrophin chimeras suggests that the presence of iPSC-utrophin in a small portion of the NMJ and of the axons is insufficient for the corrective activities.

It is possible that sarcolemma utrophin is required for the corrections. It is also possible that absence of utrophin from other compartments inside or outside the muscle plays an indirect role in the development of the skeletal muscle pathology. The early onset and rapid progression of the disease, the post-synaptic defects at the NMJ, the increase in mononuclear infiltration that results in massive fibrosis and the lack of a healthy pool of myogenic cell population [10], [41], [42] may also contribute to the lack of responsiveness of the mdx∶utr muscle to iPSC treatment. It would be intriguing to determine which are the structures (i.e. sarcolemma, NMJ, myogenic compartment, fibrosis) that mostly affect the iPSC rescue in the WT/mdx∶utr muscle.

The severe phenotype observed in mdx∶utr mice contrasts with the mild phenotype observed in mdx mice, and this difference is caused by global absence of utrophin. Thus, global loss of utrophin and the progressive deterioration of the animal health may be key contributors to the muscle phenotype of WT/mdx∶utr chimeras. Interestingly, the poor health of the mdx∶utr mice does not appear to compromise the recovery of other tissues like fat. Thus, different mdx∶utr tissues respond differently to the iPSC rescue.

It is possible that a higher degree of chimerism (i.e. WT/mdx∶utrophin chimeras with greater than 30% iPSC mosaicism) will increase dystrophin supply and lead to corrections in muscle. Nevertheless, it is clear from the studies of the mdx and mdx∶utrophin chimeric mice that the presence of utrophin in the chimeras is beneficial for the development of a healthy chimeric muscle.

### Sarcolemma-dystrophin versus -utrophin: is it a matter of choice?

In the WT sarcolemma, the presence of dystrophin precludes the activation of utrophin. In the mdx sarcolemma, the obligatory absence of dystrophin permits up-regulation of utrophin. In the mdx∶utrophin muscle, neither protein is produced. In the WT/mdx∶utrophin muscle, the iPSCs may supply: (a) dystrophin but not utrophin, (b) utrophin but not dystrophin, (c) both or (d) none. The WT iPSC-derived fibers, surrounded by mutant fibers containing neither dystrophin nor utrophin, produce (a) dystrophin but not utrophin. This indicates that the sequence: dystrophin as first choice, utrophin as second choice (in the absence of dystrophin) takes place not only in WT muscle but also in mosaic muscle containing WT iPSC-fibers. The mutant environment does not appear to modify the hierarchy of events that governs dystrophin/utrophin production. Future studies with mdx iPSCs aimed at supplying utrophin but not dystrophin will help further delineate the regulatory circuitry that exists between dystrophin and utrophin.

### Corrections of Fat

Intriguingly, iPSCs (and ESCs, [35]) appeared to effect corrections in fat, which does not express dystrophin but does express utrophin. Unlike mdx mice, which have reduced fat, we previously showed that ESC-chimeras regain normal fat mass [35]. The fat recovery depends on the presence of dystrophin, as mdx ESCs (lacking dystrophin) cannot exert corrections in muscle or fat tissues [35]. The fat recovery highlights a non-cell autonomous role of dystrophin, perhaps *via* paracrine factors that may bridge dystrophin-expressing tissues (heart, skeletal and smooth muscle) with dystrophin-non-expressing tissues (fat). The fat recovery gains significance in light of our recent finding that shows upregulation of muscular markers in the mdx and WT/mdx fat, like troponin, tropomyosin and myosin genes [35], and another recent discovery that links brown fat to muscle *via* a Myf5 common ancestor that has interconvertible properties [43], [44], [45], [46].

The process of inhibition of utrophin by dystrophin in the muscle sarcolemma does not appear to operate in the fat, because (a) dystrophin is not present in the fat, and (b) utrophin is produced in fat of mice that produce dystrophin in muscle. The latter observation indicates that dystrophin does not control utrophin expression at a distance. Importantly, the fat of mdx and mdx∶utrophin chimeras show mass recovery. The potential of iPSCs to recover fat in mdx∶utrophin contrasts with the ability to recover skeletal muscle in mdx∶utrophin. While the role of utrophin in the fat remains to be elucidated, it is clear from the present study that utrophin is not required for the recovery of fat. We can not formally rule out however that a minimal presence of utrophin supplied by the iPSC-fat or by other distant structures (i.e. iPSC-axons, -NMJ) triggers corrective activities in the fat of mdx∶utrophin chimeras.

Is there any reciprocal effect of utrophin-fat to the skeletal muscle? It is possible that the presence of utrophin in the fat may indirectly impact the chimeric skeletal muscle to partially stabilize the DGC with low dystrophin content to additionally contribute to its key role in the muscle. Because the recovered fat of the mdx∶utrophin chimeras has little utrophin, smaller contribution from the fat to the rescue of the skeletal muscle of the mdx∶utrophin chimeras may be expected.

Taken together, the results suggest that iPSC-dystrophin from a distance exerts trophic changes in the fat. The potential non-cell autonomous role for dystrophin, perhaps *via* signaling, does not appear to depend on utrophin mutation.

### Conclusion

WT murine iPSCs incorporate at low percentages but globally into mdx and mdx∶utrophin mice when the cells are injected at blastula stage. The iPSCs rescue the skeletal muscle in mdx but not in the mdx∶utrophin chimeras. An interrelated tissue that does not express dystrophin, like the fat, responds to the iPSC-based rescue in an utrophin-independent manner.

The blastocyst assay highlights an important requirement for utrophin that cannot be circumvented by a low percentage of dystrophin supply. The assay also highlights the importance of widespread delivery of dystrophin and the potential roles that dystrophin may play outside the muscle. Thus, blastocyst injection of iPSCs represents an important approach to study global mechanisms of disease corrections in DMD.

## Materials and Methods

### Induced pluripotent stem cells

(Oct4)-eGFP-marked WT mouse iPSCs were generously provided by Rudolf Jaenisch at the Whitehead Institute for Biomedical Research affiliated with Massachusetts Institute of Technology. WT mouse iPSCs cells grow in DMEM with high glucose, 15% FCS, glutamine, nonessential aminoacids, beta-mercaptoethanol, antibiotics and on SNLa76/7 STO cells, which constitutively express LIF.

### Mouse colonies

Mdx mice were purchased from Jackson lab. The colony was maintained by crossing mdx males with mdx females. Mdx∶utrophin+/− mice were generously provided by R. Grange. The colony was maintained by crossing mdx∶utrophin+/− males with mdx∶utrophin+/− females. All animal experiments were approved by the Institutional Animal Care and Use Committee (IACUC) of the University of Medicine and Dentistry of New Jersey (Animal Welfare Assurance Number: A3158-01).

### Generation of chimeric mice

3–4 week old mdx, mdx∶utrophin+/− and mdx∶utrophin−/− females were superovulated (PMSG and HCG, 5 IU, VWR) and mated with mdx∶utrophin+/− males. Blastocysts were collected at 3.5 days after mating and injected with 10–14 WT iPSCs. Injected blastocysts were then transferred into the uteri of pseudopregnant females and allowed to develop to term. Chimeric pups were initially identified by genomic PCR for the eGFP transgene in tail biopsies from 7 day-old pups. Global iPSC incorporation was later confirmed at time of sacrifice (10–12 weeks for WT/mdx∶utrophin and 12–15 weeks for WT/mdx) by genomic PCR for eGFP on tail, skeletal muscle (quadriceps), fat, liver, lung and spleen. Degree of chimerism was determined by semi-quantitative genomic PCR of the WT allele for dystrophin using DNA samples from skeletal muscle and by dystrophin IMF on heart cryosections as described below.

### Semi quantitative genomic PCR

Genomic DNA from all chimeras, WT, mdx and mdx∶utrophin controls was isolated from heart, tail, quadriceps, pectoralis and diaphragm by overnight digestion in SDS/Proteinase K buffer followed by phenol/chloroform extraction. 50 ng of genomic DNA was used in PCR with primers for genotyping the WT allele of dystrophin (Primers: forward–gtcactcagatagttgaagccatttag & reverse– catagttattaatgcatagatattcag). 2 ng of the same DNA was used in PCR with Id1 [47] and GAPDH control primers (Clontech) for internal control. PCR was performed with puReTaq Ready-To-Go PCR beads (GE Healthcare) in the log phase (using 28–35 cycles). Scans were performed on Typhoon 8600 Variable Mode Imager (Molecular Dynamics), and quantitation done using ImageQuant [35].

### Histology, IMF, and Western Blot (WB)

IMF for dystrophin (1∶20, dys2 Novocastra), utrophin (1∶1000, generously supplied by Dr. Fritschy, University of Zurich), Dbv-alpha (1∶200, 610766, BD Transduction Laboratories), syntrophin (1∶100, MA1-745, ABR) and alpha-bungarotoxin (1∶250, Invitrogen) was performed on 10 µm thick cryosections of heart and diaphragm. Nuclei were identified with DAPI (Vector Labs).

WB was performed using antibodies reactive with dystrophin (dys1, Novocastra), utrophin (MANCHO3, clone8a4s, Developmental Studies Hybridoma Bank), dystrobrevin-alpha and syntrophin. A 4–20% gradient acrylamide gel and high molecular weight protein standards (HiMark, Invitrogen and Precision Plus, Biolab) were used. The intensities of the WB exposures were quantified using Quantity One software on a GS800-Densitometer (Bio-Rad). The relative expression levels were normalized using tubulin bands within the same linear range of detection. Visualization of fibrosis was performed using a Masson Trichrome stain kit (Richard-Allan Scientific) on 6 µm thick paraffin-embedded sections of heart, quadriceps and diaphragm. H&E was also performed on paraffin sections of heart, quadriceps and diaphragm to visualize cell morphology and pathology.

### Functional analysis

12-week old WT/mdx, mdx, and WT animals were deeply anesthetized (2 mg xylazine-20 mg ketamine/100 g body mass ip). Both fast-twitch extensor digitorum longus (EDL) muscles were excised and incubated at 30°C in an oxygenated (95% O2–5% CO2) physiological salt solution (PSS; pH 7.6) containing (in mM) 120.5 NaCl, 4.8 KCl, 1.2 MgSO4, 20.4 NaHCO3, 1.6 CaCl2, 1.2 NaH2PO4, 10.0 dextrose, and 1.0 pyruvate. Silk suture (4-O) was tied to the distal and proximal tendons of the EDL at the myotendinous junctions. Muscles were then fixed between a clamp at the base of the bath and arm of a dual-mode servomotor system (300B, Aurora Scientific), or an isometric force transducer (Grass FT03), at a resting tension (L0) of 1.0 g. EDL muscles were maintained at L0 by a stepper motor [48]. The servomotor arm, stepper motor and electrical stimulator were controlled by Dynamic Muscle Control software (DMC Version 4.1.6, Aurora Scientific) to obtain isometric force data. Isometric twitch and tetanic data were obtained. Length of the muscles was obtained with a micrometer; each muscle was weighed to the nearest 0.1 mg using an A-200D electronic analytical balance (Denver Instruments, Denver, Colorado) and then muscles were snap frozen in liquid nitrogen for subsequent sectioning and WB analysis. Muscle cross-sectional area (CSA) was determined as previously described [49]. Twitch and tetanic forces were normalized to muscle CSA to obtain twitch and tetanic stress (g/mm2). As every muscle has a distinct pattern of iPSC incorporation in a chimera, the values obtained from the 2 EDL muscles from the same WT/mdx animal (n = 2) were independently considered, and thus, n = 4 per WT/mdx group [35].

### Data analysis

Results are presented as mean ± s.e.m or as a range. Statistical comparison was performed with nonparametric two-tailed unpaired analysis of variance. A probability value of <0.05 was considered to be statistically significant.
